# Editorial: Artificial intelligence in animal behaviour, veterinary behaviour and neurology

**DOI:** 10.3389/fvets.2025.1607623

**Published:** 2025-06-24

**Authors:** Jasmin Nicole Nessler

**Affiliations:** Foundation Clinic for Small Animals, Neurology and Neurosurgery Service, University of Veterinary Medicine Hannover, Hanover, Germany

**Keywords:** artificial intelligence, machine learning (ML), deep learning, behavior, veterinary neurology

Recent technological breakthroughs—ranging from computer vision and sensor-based analytics to robotics and large language models—are transforming veterinary science. This Research Topic brings together nine studies illustrating how these innovations can address both longstanding and emerging challenges in animal health, behavior, and welfare.

A central theme in several of the contributions is precision livestock management. One study proposed a three-phase monitoring system that integrates a multi-part detection network for flock inventory, a facial classification model for identity recognition, and a facial expression analysis network for health assessment (Zhang, Zhao et al.). By refining deep learning architectures, the authors achieved marked gains in detection accuracy and efficiency, underlining how targeted enhancements such as multi-link convolution fusion blocks and re-parameterizable convolution can elevate both performance and speed. Their framework exemplifies how machine learning (ML) solutions can help farmers more efficiently monitor livestock in large-scale or complex settings, potentially reducing labor requirements and supporting better animal welfare outcomes.

Several articles in this Research Topic highlight the synergy between bioinspired engineering and animal-based research. One study, motivated by goose neck biomechanics, designed a bionic robotic arm that prioritizes both flexibility and bearing capacity in confined spaces (Zhang, Sun et al.). By systematically analyzing the goose neck's movements in narrow environments, the authors derived parameters that could inform more advanced and adaptive robotic systems. Their approach bridges animal physiology with practical robotics, opening new possibilities for applications in veterinary procedures, agritech, and beyond.

While precision livestock approaches often focus on optimizing herd management, another cluster of papers underscores how machine learning augments veterinary diagnostics and clinical practice. A large-scale study demonstrated that Bayesian Networks and Random Forests can accurately predict structural epilepsy in dogs (Flegel et al.). This approach, validated through tens of thousands of ML models, showcases how data-driven methods can strengthen clinical decision-making. The authors' success in isolating features highly predictive of structural epilepsy paves the way for improved diagnostic protocols, potentially reducing uncertainties for both clinicians and pet owners.

Expanding on these applications of ML, other researchers explored automated approaches in canine science, reflecting the growing need to analyze complex, high-volume behavioral data (Farhat et al.). By systematically reviewing existing literature and conducting empirical work with animal behavior researchers, they pinpointed both the promise and challenges of automation. Their recommendations could lead to greater acceptance of automated methods, ultimately promoting more robust and reproducible behavioral studies in companion animals.

Meanwhile, in an investigation of grazing behaviors on the Qinghai-Tibetan Plateau, researchers combined field observations with meta-analysis to uncover how temporal and spatial factors shape yak foraging patterns (Yang et al.). Their findings highlight how local climate conditions, forage quality, and livestock traits intertwine to influence grazing behaviors. Such insights can help refine herd management practices, ensuring that rangelands remain sustainably productive under changing environmental pressures.

A complementary line of research explores non-invasive indicators of cattle welfare, specifically vocalization patterns under negative affective states (Gavojdian et al.). By categorizing low- and high-frequency calls, the authors developed deep learning and explainable machine learning frameworks to identify individual cows and gauge stress responses. This work reinforces the importance of acoustic monitoring as part of a precision livestock farming toolkit, offering a tangible route toward earlier detection of distress or discomfort in herd animals.

Another study tackled the early detection and prediction of digital dermatitis—a prevalent cause of lameness in dairy herds—using sensor-based ML models (Magana et al.). The application of automated pipelines, including the Tree-Based Pipeline Optimization Tool (TPOT), shows promise for alerting farmers to emerging disease outbreaks before clinical signs fully manifest. By leveraging subtle shifts in cow behavior, these models could lead to timely interventions that improve overall herd health and productivity.

As these data-driven methods proliferate, the role of generative AI has become a topic of both excitement and caution. One team posed the question: “*Can ChatGPT diagnose my collapsing dog?*” and delved into how large language models might intersect with veterinary triage or diagnostics (Abani, De Decker et al.). Their findings underscore the need for critical evaluation of AI-suggested “diagnoses,” given that statistical pattern-matching differs fundamentally from true clinical reasoning. In a related article, the authors examined ChatGPT's potential influences on research in veterinary neurology (Abani, Volk et al.). While acknowledging the efficiencies in literature exploration, they also highlight ethical concerns, such as overreliance on AI-generated texts or the spread of misinformation.

Collectively, these nine contributions exemplify how innovation in artificial intelligence, robotics, and data analytics can reshape the practice of veterinary science, from farm management and clinical diagnostics to animal welfare and fundamental research. While each study addresses a distinct question, they converge on a shared principle: harnessing technology to generate timely, actionable insights that benefit both animals and the humans who care for them. Yet, these advancements are not without challenges: ensuring data quality, building transparent models, and maintaining ethical oversight remain vital to the responsible adoption of such tools.

We hope this Research Topic serves as a stepping stone for cross-disciplinary collaboration. Whether you are a veterinarian, researcher, engineer, or policy-maker, the findings outlined here illustrate the rapid evolution of veterinary science in our data-driven era. By embracing these methods while engaging in thoughtful discourse on their limitations, we can collectively advance a more sustainable, innovative, and compassionate future for animal care.

## Author's personal thoughts on this editorial

This editorial was created by a large language model (LLM) (1) with the prompt: “Write an editorial... (see acknowledgments below)”. No additional revisions were made on the created text. While good human writing is often characterized by an engaging narrative style and storytelling – even in scientific publications this editorial feels rather flat: While the information extracted from the abstracts provided to the LLM is accurately conveyed and presented in highly readable language, a thematic summary of related abstracts, with a more integrative discussion and a take-home-message would be preferable for a well-crafted editorial. It appears that outstanding writing still relies on the human ability to bring depth and new ideas to the narrative.
